# Optimizing the Application Timing and Dosage of *Metarhizium brunneum* (Hypocreales: Clavicipitaceae) as a Biological Control Agent of *Aedes aegypti* (Diptera: Culicidae) Larvae

**DOI:** 10.1093/jme/tjac186

**Published:** 2022-12-20

**Authors:** A M Alkhaibari, M J Wood, S I Yavasoglu, J C Bull, T M Butt

**Affiliations:** Department of Biology, Faculty of Science, University of Tabuk, Tabuk 71491, KSA; Department of Biosciences, Faculty of Science and Engineering, Swansea University, Singleton Park, Swansea SA2 8PP, UK; Department of Biology, Faculty of Science, Aydın Adnan Menderes University, 09100, Aydın, Turkiye; Department of Biosciences, Faculty of Science and Engineering, Swansea University, Singleton Park, Swansea SA2 8PP, UK; Department of Biosciences, Faculty of Science and Engineering, Swansea University, Singleton Park, Swansea SA2 8PP, UK

**Keywords:** *Metarhizium brunneum*, *Aedes aegypti*, larvae, dynamics, control

## Abstract

*Aedes aeg*ypti (Diptera: Culicidae) is the principal vector of dengue and other viruses that cause disease among 100 to 400 million people each year. The recent development of widespread insecticidal resistance has led to the rapid development of biological control solutions aimed at larval control. While the efficacy of *Metarhizium brunneum* has been shown against *Aedes* larvae, the impact of larval population dynamics will need to be determined to formulate effective control strategies. In this study, larvae were subjected to four concentrations of *M. brunneum* (10^5^, 10^6^, 10^7^, 10^8^ conidia ml^−1^). Larvae were found to be susceptible to *M. brunneum* with dose-dependent efficacy. When constant larval immigration was added as a parameter, peak mortality was consistently found to occur on the fourth day, before a significant reduction in control efficacy linked to a decline in conidial availability within the water column. This suggests that *M. brunneum* treatments should be applied at a concentration 1 × 10^7^ conidia ml^−1^ every four days to effectively control mosquito larvae in the field, regardless of the fungal formulation, water volume, or larval density. Understanding fungal-mosquito dynamics is critical in developing appropriate control programs as it helps optimize the fungal control agent’s dose and frequency of application.

*Aedes aegypti* (Diptera: Culicidae) is a widely distributed vector of dengue, chikungunya, yellow fever, zika, and other harmful arboviruses which are known to have a devastating impact on human health (Leta et al. 2018). Currently, more than half of the world’s population is at risk from such diseases. The geographic range of *Ae. aegypti* and *Ae. albopictus* mosquitoes has significantly increased due to factors including climate change, increased trade, anthropogenic activity, and the ability of immatures stages of these species to utilize a wide range of breeding habitats (Bennett et al. 2019, Ryan et al. 2019, Brugueras et al. 2020, Müller et al. 2020). Control methods, aimed at preventing population growth and spread, are often hampered by the cryptic developmental sites utilized by *Ae. aegypti* and *Ae. Albopictus* mosquitoes utilize. They are frequently found near residential properties; ovipositing in water butts, plastic buckets, and other artificial containers; alongside less obvious sites such as bottle tops, temporary water pools or refuse (Parker et al. 2020, Mullin 2021). *Aedes* females select suitable breeding sites based on their physicochemical characteristics. To enhance the development and survival of their offspring, they lay their eggs in sites that reduce exposure to predators (Pamplona et al. 2009), parasites (Zahiri et al. 1997), and competitors (Chadee et al. 1990, Zahiri and Rau 1998, Seenivasagan et al. 2009) or increase access to food (Allan and Kline 1995, Ponnusamy et al. 2008). Wong et al (2011) reported that, *Ae. aegypti* females are more likely to lay their eggs in containers already inhabited by conspecific immatures. Oviposition behavior of *Aedes* mosquitoes inclusive of their ability to utilize cryptic, often transient habitats, results in highly fractured spatial population distributions. It also creates a major challenge for public health authorities relating to the design of appropriate control measures (Caputo et al. 2012).

Pyrethroid insecticides have historically provided effective control of adult mosquitoes, however, these chemicals are highly toxic to aquatic invertebrates, fish, and other organisms, while also posing a potential public health risk; rendering them unsuitable as larval control solutions (Bonds 2012). Furthermore, widespread insecticidal resistance is commonplace as a result of the extensive use of these pesticides, inclusive of the larvicide propoxur (Faucon et al. 2017, Dusfour et al. 2019, Sierra et al. 2021). Therefore, there is an urgent global requirement to develop sustainable, environmentally friendly biological control agents for mosquito control.

Effective biological control agents for mosquito larval control include microbes, such as *Bacillus thurungiensis subsp. israelensis* and *Metarhizium brunnuem,* alongside augmentative introductions of naturally occurring predators (Shaalan and Canyon 2009, Greenfield et al. 2015, Becker and Lüthy 2017, Oliveira et al. 2017, Brühl et al. 2020). While field efficacy has been demonstrated to some extent, cost and control stability have prohibited widespread adoption of these methods. Entomopathogenic fungi (EPF) such as *Beauveria bassiana* and *Metarhizium brunneum* (Hypocreales: Clavicipitaceae), which kill all life stages (eggs, larvae, pupae, adults) of *Aedes* and other mosquito species (Alkhaibari et al. 2016, Flor-Weiler et al. 2017, Carolino et al. 2019, Rodrigues et al. 2019), rank among the most promising biological solutions since they are relatively inexpensive and are efficacious in disparate environments.

EPF efficacy is dependent on several factors including virulence of the fungal strain, spore form (conidia or blastospores), dose, formulation, and carrier (Alkhaibari et al. 2017, Carolino et al. 2019, Loureiro et al. 2019). Both the blastospores and conidia of *M. brunneum* are effective in killing *Aedes* larvae, with blastospores being marginally more virulent (Alkhaibari et al. 2017). Liquid suspensions of *Metarhizium* conidia appear to work better as larvicides than dry-spore products, both in laboratory and field studies (Kamalakannan and Murugan 2011), albeit with differing modes of action (Lacey et al. 1988, Butt et al. 2013). Mortality is dose-dependent with higher doses of conidia causing rapid and maximal control of mosquito larvae (Greenfield et al. 2015). Larval density has, however, proven to be a complicating factor in terms of EPF dosage efficacy; EPF are less efficacious at high larval densities, presumably due to dilution of the inoculum relative to the target individuals (Pelizza et al. 2007).

Application rates not only influence mosquito control efficacy, but also end-user cost. It is therefore important to optimize the application rate (dose and frequency) to ensure maximal control. To date, there are very few studies on this topic. Most EPF are typically sprayed using a rate of 1 × 10^8^ conidia ml^−1^ to adhere to prior recommendations (Bukhari et al. 2011, Kamalakannan and Murugan 2011). Whatever the dose, however, the amount of viable inoculum will gradually decline over time due to a range of climatic and biotic factors. Conidia are likely to become diluted within the water column; sedimentation, drift, and nontarget feeding activity all serve to reduce spore availability for mosquito larval control (Scholte et al. 2004). More important than conidial viability, however, is the quantity and state of spore bound proteases, especially Pr1 which triggers stress induced mortality in *Aedes* larvae (Butt et al. 2013).

This study investigates the susceptibility of *Ae. aegypti* larvae to conidia of *M. brunneum* whilst larval population dynamics are altered and adjusted to simulate natural fluctuations exhibited in typical breeding grounds. Constant oviposition (“immigration”) and replenishment of infested water bodies with *Ae. aegypti* larvae are simulated to provide information on the key determinants for effective application. The findings of this study provide an understanding that outlines the effects of larval population dynamics on EPF efficacy, providing enhanced guidance on dosage and application frequency requirements.

## Methods and Materials

### *Aedes aegypti* Maintenance

*Aedes aegypti* (strain AeAe) eggs, obtained from the London School of Hygiene and Tropical Medicine (UK), were hatched in tap water in 2.5 liter square plastic tubs (23 × 23 cm at top) within bugdorms (50 × 50 × 50 cm). Larvae were fed on crushed rabbit food pellets (Burgess, UK) and kept at 25 ± 2°C in a 16:8 (L:D) photoperiod controlled-temperature room.

### Fungal Strains and Production

*Metarhizium brunneum* isolate ARSEF 4556 (origin: *Boophilus* sp., USA), identified as highly pathogenic to *Aedes* mosquitoes (Greenfield et al. 2015), was maintained on Sabouraud dextrose agar at 27 ± 1°C for 15 d. Conidia used in assays had over 95% viability as determined by the plate-count technique (Goettel and Inglis 1997). Conidial concentrations were determined using an improved Neubauer hemocytometer.

### Susceptibility of *Ae. aegypti* Larvae to *M. brunneum* (Strain: ARSEF 4556)

Baseline susceptibility of *Ae. aegypti* larvae was determined in a series of bioassay experiments using both ‘wet’ and ‘dry’ formulated *M. brunneum* conidia (strain: ARSEF 4556) at four different concentrations (1 × 10^5^, 1 × 10^6^, 1 × 10^7^, and 1 × 10^8^ conidia ml^−1^). *M. brunneum* was applied either as a ‘dry’ conidial application at the water’s surface or a ‘wet’ formulated product suspended in 0.03% aqueous Tween 80 (Sigma Aldrich, MI) to assess control efficacy for each application type.

Fifteen *Ae. aegypti* larvae (L_4_) were placed into round plastic containers (92 mm. width, 250 ml capacity) in 200 ml distilled water. A Suspension of ‘wet’ *M. brunneum* or an even dusting of ‘dry’ conidia was applied to the water to give the desired concentrations. Control larvae were exposed to 0.03% Tween 80 solution only or plain distilled water. Larval mortality was recorded every 24 hr for a total of six days before experiments were terminated. Each treatment was replicated three times and the whole experiment was repeated three times (*n* = 135 per treatment = 15 larvae per assay × 3 replicates × 3 repeats total).

### Constant Density

Further experiments were conducted to determine the cause of decreasing mortality over time in ‘larval density’ assays. Ten *Aedes aegypti* larvae (L_4_) were placed into round plastic containers (92 mm. width, 250 ml capacity) filled with 150 ml of distilled water. Wet formulated *M. brunneum* conidia were applied to give a concentration of 1 × 10^7^ conidia ml^−1^. Treatments were assessed at each 24-hr period, at each time point dead larvae were counted and removed before an equal number of new larvae were introduced to maintain a constant density over the course of a six-day experiment. Controls consisted of distilled water treated with 0.03% Tween 80 only.

### Constant Immigration

The aim of this experiment was to monitor changes in mortality over time as larval density increases, simulating constant oviposition within the water body to address whether sufficient inoculum would remain available to cope with increasing larval densities.

Plastic containers (95 mm width, 280 ml volume or 69 mm width, 155 ml volume) were filled with three volumes of distilled water; 50l, 100, and 250 ml. Larvae of *Ae. Aegypti* (L_4_, *n* = 10) in three replicates were added to each container and conidia of *M. brunneum* (1 × 10^7^ conidia ml^−1^) were applied to the surface of as either ‘dry’ spore formulations or ‘wet’ suspensions in 0.03% aqueous Tween 80. Control experiments consisted of distilled water treated with 0.03% Tween 80 only (50, 100, and 250 ml). After each 24 hr period, dead larvae were counted and removed, before 10 additional larvae were introduced to simulate constant larval immigration for a total elapsed time of six days.

### Statistical Analysis

Survival data were graphically presented as Kaplan-Meier plots, using the ‘survminer’ R package. Survival regression was performed to test hypotheses on the effects of ‘wet’ or ‘dry’ treatment and dose, using the ‘survival’ R package. Dose was fitted as a categorical variable to accommodate nonlinear relationships. Individuals that survived beyond the duration of the trial were classed as right censored.

In trials where further larvae were added to trials, proportional survival was modeled using β-regression and absolute survival was modeled using generalized additive models (GAMs) with quasi-Poisson error structure. This is an analysis of covariance, performed through the fitting of a β-regression model of survival proportion, with time as a continuous explanatory variable and treatment as a categorical explanatory variable. All statistical analyses were performed using R version 4.0.5 (R Core Team 2021).

## Results

### Susceptibility of *Ae. aegypti* Larvae to *Metarhizium brunneum* (Strain: ARSEF 4556)

**Fig. 1.**
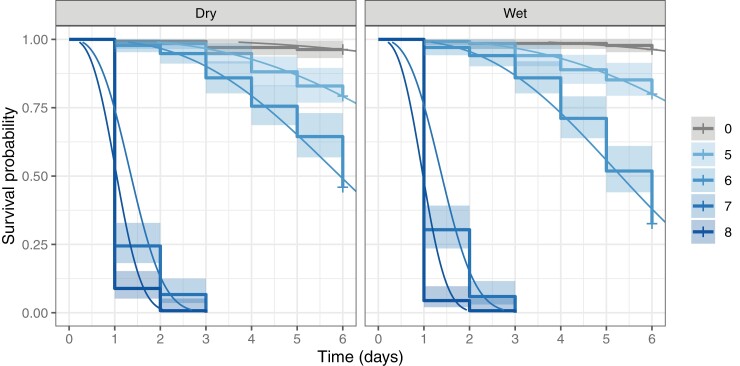
Kaplan-Meier survival plots for cohorts of *Aedes aegypti* larvae following treatment with ‘dry’ or ‘wet’ *Metarhizium brunneum* (ARSEF 4556) conidia at four concentrations (color-coded as log_10_-transformed conidia ml^−1^) and a blank control (‘0”’). Fitted curves show predicted survival based on parametric survival regression and 95% confidence intervals shown as shaded ribbon.

Initial bioassays demonstrate the susceptibility of *Ae. aegypti* larvae to both ‘dry’ and ‘wet’ formulations of *M. brunneum* (Strain: ARSEF 4556). The differences between ‘wet’ and ‘dry’ were not significant (Χ^2^_df = 4_ = 4.38, *p* = 0.36, survival regression), however, the effect of dose on larval mortality was significant (Χ^2^_df = 4_ = 2431, *p* < 0.001, survival regression). Applied concentrations of 1 × 10^8^ and 1 × 10^7^ conidia ml^−1^ resulted in rapid mortality within three days, with 1 × 10^8^ conidia ml^−1^ eliciting the most rapid response, generally reaching 100% mortality within 48 hr. Applications of 1 × 10^6^ and 1 × 10^5^ conidia ml^−1^ for both ‘wet’ and ‘dry’ formulations were substantially less effective than higher concentrations, but mortality was still seen to be higher than that of the controls (Fig. 1).

### Constant Density Trials

**Fig. 2.**
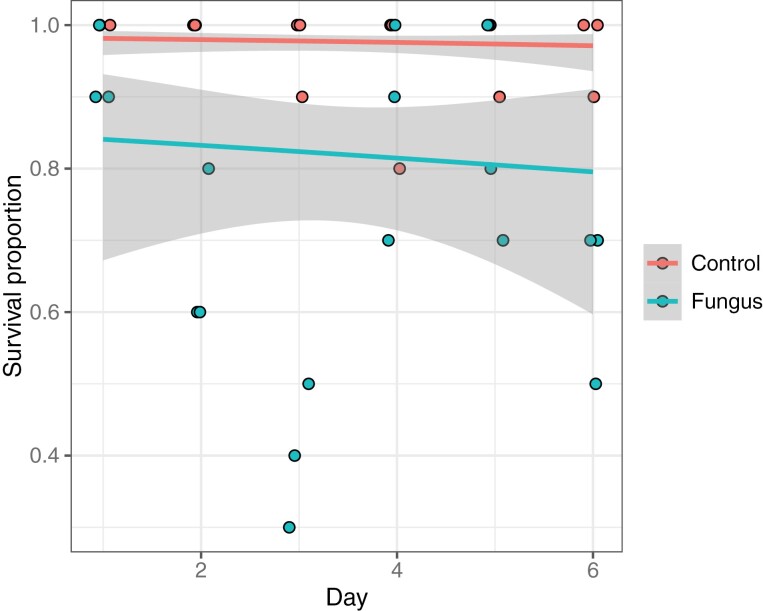
Proportional survival of *Aedes aegypti* larvae following treatment with ‘wet’ *Metarhizium brunneum* (ARSEF 4556) conidia at 1 × 10^7^ conidia ml^−1^ in 150 ml volume. Red indicates control larvae; green indicates treated larvae. Grey ribbons indicate 95% confidence intervals from a fitted β-regression. A small amount of horizontal ‘jitter’ was applied to data points to uncover points recorded on the same day.

Having established there was no difference in survival between ‘dry’ and ‘wet’ conidial preparations, we quantified the effect of *M. brunneum* treatment under varied simulated environmental conditions. When larval populations were supplemented to simulate immigration into an environment that would maintain constant carrying density (Fig. 2), there was a reduction in survival between conidia-treated and untreated control populations (Χ^2^_df = 1_ = 14.9, *p* < 0.001, β-regression), but no statistically significant change in survival over time (Χ^2^_df = 1_ = 0.744, *p* = 0.388, β-regression).

### Constant Immigration Trials

**Fig. 3.**
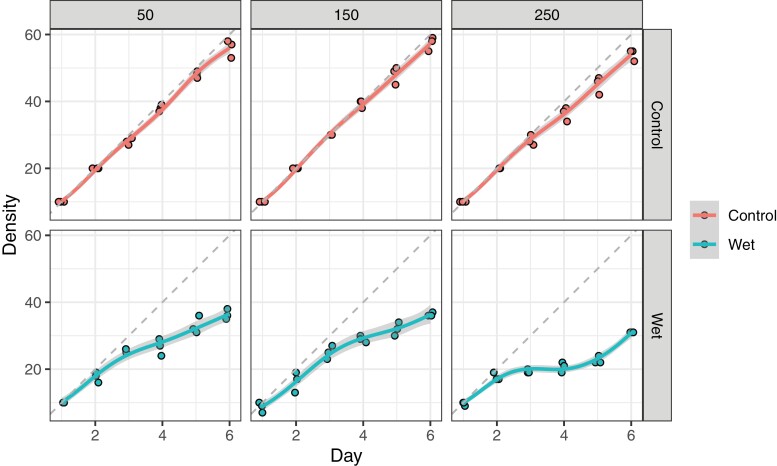
Numbers of surviving *Aedes aegypti* larvae following treatment with ‘wet’ *Metarhizium brunneum* (ARSEF 4556) conidia at 1 × 10^7^ conidia ml^−1^ in 50, 150, and 250 ml volumes. Red indicates control larvae; green indicates treated larvae. Grey ribbons indicate 95% confidence intervals from a fitted generalized additive model. A small amount of horizontal ‘jitter’ was applied to data points to uncover points recorded on the same day. Dashed diagonal lines show theoretical population increase with no mortality.

To explore the alternative scenario, whereby mosquito larval populations vary according to constant natural immigration and larval death, a constant larval immigration rate was applied to the experiment (Fig. 3). Statistically significant reductions in larval population size between fungus-treated and control populations were observed (Χ^2^_df = 3.7_ = 61.6, *p* < 0.001, quasi-Poisson GAM). Intriguingly, pronounced reductions in mortality rate amongst treated larvae were observed towards the end of the trial (Fig. 3). This trend was significantly stronger when water volumes were increased (Χ^2^_df = 7.2_ = 9.24, *p* < 0.001, quasi-Poisson GAM).

## Discussion

To date, several research groups have demonstrated the efficacy of both blastospore and conidial formulations of *M. brunneu*m for mosquito larval control (Fakoorziba et al. 2014, Greenfield et al. 2015, Alkhaibari et al. 2016). This study adds further evidence to support their efficacy as larvicidal biocontrol agents, whilst providing new insight into application requirements in relation to domestic *Aedes* populations using containers as immature development sites. All parameters altered to simulate varied larval population dynamics resulted in peak mortality at the fourth day, after which point, efficacy rapidly declined. Densities of experimental *Aedes* larvae were varied during the ‘constant immigration’ trials; that the general trend of peak mortality on the fourth day described above was seen to be consistent in these trials suggests that, generally speaking, larval mortality may be independent of fungal efficacy. This trend may be explained by the availability of viable conidia within the water column, higher availability may lead to increased mortality, whereas substrate settling of the conidia after a few days would reduce availability, conferring lower mortality.

Formulation and application methodology may also be used to increase larval mortality following field application with the fungus. Dry spore applications would be more susceptible to external environmental factors including heat and UV which are known to negatively influence fungal stability (Braga et al. 2007, de Freitas et al. 2014). In these instances, oil formulations may offer some protection from these abiotic factors; neem-oil formulation, for instance, has been shown to protect the spores from UV exposure for up to two hours (Paula et al. 2019). This effect is, however, short lived, meaning that practically surface deployments may be seen as less effective. Additionally, the oils may affect air breathing beneficial organisms present within the water column, meaning that sub-surface application would likely be preferred (Lee et al. 2018)

Typical ‘wet’ formulations offer the conidia some level of protection from environment, but are susceptible to sedimentation and osmotic rupturing postinoculation (Hegedus and Khachatourians 1995). In these instances, consideration of larval behavior will be important. Sedimentation effects would be of primary concern for the control of *Anopheles* and *Culex* species, both of which are known surface and water column filter feeders; for *Aedes* species this may be less disadvantageous given that they are preferential browsers, feeding on settled particulate matter on substrates (Dahl et al. 1988, Merritt et al. 1992, Workman and Walton 2003). Sedimentation and cell rupturing would have to be mitigated through repeat applications of the control agent at four-day intervals for high control efficacy.

Conidia sedimented within 24 hr, with the majority of conidia available to larvae on the bottom of the experimental arenas. Larvae were seen to ingest these conidia, as would be expected for substrate browsers such as *Aedes aegypti* (Dahl et al. 1988, Merritt et al. 1992, Workman and Walton 2003). This consumption was however minimal and would not affect spore availability, however, those available spores may have reduced pathogenicity as a result of PR1 ‘washing’. ‘Washing’ is a described effect of formulating entomopathogenic conidia in aqueous solutions whereby extracellular proteinases are hydrolytically washed from the spore surface (Shah et al. 2005). This effect may further explain the drop in conidial virulence, should PR1, a molecule known to confer fungal pathogenicity, be removed from the spore surface over time, a correlated drop in pathogenicity would be expected as seen in the described experiments. Butt et al. (2013) demonstrated the influence of PR1 on larval mortality as a primary cause of stress related mortality in aquatic conditions, ‘washing’ of this proteinase from the cell wall of the fungi may therefore severely impact the longevity of the conidia. Additionally, continued production of PR1 would likely be affected by the pH of the water, given that PR1 is only actively produced under alkaline conditions at around pH 8 (St. Leger et al. 1998). Genetic manipulation of the strains used for application may therefore prove useful, if cost-ineffective, way of regulating high-PR1 production, or indeed increased production, postapplication. Additional gene manipulation in such a manner may also allow for expression of other ‘foreign’ toxins that would improve performance and longevity. Alternatively, specific strain selection could be used to optimize treatments; profiling of different strains capacity to produce PR1, and the length of time for which they would do so, would allow for careful selection leading to potential increases in mortality over time as PR1 is directly correlated to virulence (Shah et al. 2007). Once efficacious strains have been selected, careful culture management can be used to enhance PR1 production and infer greater virulence in products destined for management practices.

To sustain larval mortality, while simultaneously mitigating the cost of regular applications; integrated semiochemical deployment may offer potential enhancements to the strategy. *Aedes* mosquitoes frequently oviposit in sites with prior *Aedes* infestation, responding to larval secretory compounds such as heneicosane (Mendki et al. 2000). Oviposition attractants have the potential to be used to attract gravid females to traps or specific sites inoculated with a control agent as part of a ‘lure and kill’ strategy (Ong and Jaal 2015, Hapairai et al. 2021), reducing the treatment area and, therefore the total application requirement. Another option to spread out the availability of conidia to larvae over time, would be fungal dispersal mediated through bait stations. Sugar-based baits containing pyriproxifen have been shown to be effective for control of adult and larval *Aedes albopictus* when applied to plants (Fulcher et al. 2014). The same ‘lure and kill’ principle may be applicable to aquatic larvae should suitably feed baits be mixed with fungal spores and deployed over time. Such a strategy may be implemented using commercial products such as fishing ‘donks’, commercial products widely available in the USA which are used as fish feeders. Versions of these products could be adapted to dispense larval foodstuffs treated with *Metarhizium* spores. These baits would be gradually released over time, mitigating for rapid pathogenic declines due to conidial immersion in the water column. While such a strategy could be used to reduce application requirements over time, further work will be required to assess the effect of such systems within the larger framework of local population dynamics.

This study demonstrates that EPF applications will need careful consideration of the target organisms’ population dynamics. Effective control may be achieved, if EPF are consistently applied at a rate of once every four days; after which point the availability and viability of the conidia suffer significant decreases within the water column. Formulations in novel materials, including oil formulations and bait dispensers, may help to alleviate these issues, decreasing the application rate requirements and further enhancing total control levels over time.
